# Understanding Insulin Resistance in NAFLD: A Systematic Review and Meta-Analysis Focused on HOMA-IR in South Asians

**DOI:** 10.7759/cureus.70768

**Published:** 2024-10-03

**Authors:** Margeyi Mehta, Jigish Shah, Urvish Joshi

**Affiliations:** 1 Department of Clinical Biochemistry, Government Medical College and Sir Sayajirao General (SSG) Hospital, Vadodara, IND; 2 Department of Microbiology, Pratham Microbiology Laboratory, Vadodara, IND; 3 Department of Community Medicine, Narendra Modi Medical College and Lallubhai Gordhandas (LG) Hospital, Ahmedabad, IND

**Keywords:** non-alcoholic fatty liver disease (nafld), homeostasis model assessment of insulin resistance (homa-ir), pathology and clinical biochemistry, south asian, insulin resistance indexes, metabolic-associated fatty liver disease (mafld), homa-ir, nafld

## Abstract

Non-alcoholic fatty liver disease (NAFLD), a metabolic condition, is becoming increasingly common in South Asia. While its clinical diagnosis primarily relies on sonography and altered hepatic biomarkers, the significance of non-hepatic indicators, such as Homeostasis Model Assessment of Insulin Resistance (HOMA-IR), in relation to NAFLD requires further examination in the South Asian population due to ethnic differences in these markers. This study examined the relationship between insulin resistance, quantified using the Homeostasis Model Assessment of Insulin Resistance (HOMA-IR), and NAFLD, along with other non-hepatic biomarkers.

A thorough literature review was conducted in accordance with the Preferred Reporting Items for Systematic Reviews and Meta-Analyses (PRISMA) guidelines. We searched the PubMed, Embase, and Google Scholar databases, yielding 287 articles. After applying the selection criteria and screening, 22 studies were selected for inclusion in the analysis. We extracted and meta-analyzed the data on HOMA-IR in patients with NAFLD, along with other relevant parameters. The Newcastle-Ottawa Scale (NOS) was used to assess the quality of observational studies, whereas the RoB 2.0 tool was employed for randomized controlled trials (RCTs). The systematic review uncovered that individuals with NAFLD demonstrated statistically significant elevations in HOMA-IR levels, with a weighted mean difference (WMD) of 1.28 (95% confidence interval (CI): 1.00-1.58, I² = 98%, p < 0.0001) when compared to healthy subjects. Additionally, NAFLD patients showed markedly higher fasting blood glucose (FBG) levels, with a combined mean difference of 15.64 mg/dL (95% CI: 11.03-20.25, I² = 92%, p < 0.0001). The analysis also revealed increased triglyceride levels in NAFLD patients, with a pooled mean difference of 42.49 mg/dL (95% CI: 29.07-55.91, I² = 97%, p < 0.0001), and elevated C-reactive protein (CRP) levels, with a pooled mean difference of 2.17 mg/L (95% CI: 2.01-2.33, I² = 23%, p < 0.0001). Interestingly, subgroup analysis indicated that obese NAFLD patients exhibited significantly higher HOMA-IR levels than their non-obese counterparts, with a weighted mean difference of 5.85 (95% CI: 4.88-6.81, I² = 0%, p < 0.0001). Variations in study methodology, diagnostic techniques, and subject demographics were identified as sources of heterogeneity. The analysis found little evidence of publication bias, which lends credibility to the results. In South Asian populations, higher HOMA-IR, triglyceride-glucose (TyG) index, and CRP levels are associated with an increased risk of NAFLD. To improve the understanding and treatment of NAFLD in this specific demographic group, it is necessary to establish uniform diagnostic criteria and conduct additional studies, particularly RCTs.

## Introduction and background

Metabolic dysfunction-associated fatty liver disease (MAFLD), also referred to as non-alcoholic fatty liver disease (NAFLD), is a metabolic disorder characterized by excessive fat accumulation in the liver. This condition, which is strongly associated with insulin resistance and metabolic syndrome, has emerged as a major global health issue. The prevalence of NAFLD/MAFLD has risen significantly, affecting approximately 25% of individuals worldwide. In South Asian countries such as India and Sri Lanka, the incidence ranges from 24% to 32.74%, with urban areas in India experiencing rates as high as 53% due to lifestyle-related factors [1,2]. Ultrasonography (USG) serves as the primary diagnostic tool for NAFLD/MAFLD, often complemented by abnormal liver biomarkers detected in blood tests. Additionally, non-hepatic biomarkers, particularly Homeostasis Model Assessment of Insulin Resistance (HOMA-IR), play a crucial role in diagnosis, given that insulin resistance is fundamental to the development of NAFLD/MAFLD [2,3].

HOMA-IR is calculated by multiplying fasting insulin (µU/mL) by fasting glucose (mmol/L) and dividing by 22.5 or by 405 if glucose is in mg/dL. Healthy individuals typically score between 1 and 2, with higher values indicating greater resistance [2,3]. Recent guidelines recommend HOMA-IR for identifying metabolic disorder risks and guiding interventions [2,3]. HOMA-IR is crucial in assessing insulin resistance in metabolic syndrome, diabetes, and liver diseases. Current recommendations suggest using HOMA-IR to assess risks of metabolic disorders and determine appropriate interventions. NAFLD diagnosis requires detecting hepatic steatosis via imaging such as USG or histology while excluding significant alcohol intake and other liver conditions. The American Association for the Study of Liver Diseases (AASLD) advises assessing metabolic risk factors alongside NAFLD diagnosis [4].

Ethnic differences influenced by genetic, environmental, and lifestyle factors complicate the understanding of HOMA-IR. Iranian and Egyptian populations exhibit varying HOMA-IR thresholds, emphasizing the necessity for ethnicity-specific benchmarks [5,6]. Mexican Americans demonstrate genetic predispositions to different HOMA-IR levels associated with cardiovascular risks, suggesting that a universal approach may be insufficient [7]. The Brazilian Metabolic Syndrome Study underscores the importance of region-specific clinical guidelines, as HOMA-IR cut-offs in Brazil differ from those in Western populations [8]. Global literature demonstrates a strong correlation between hepatic biomarkers in NAFLD and non-hepatic indicators such as C-reactive protein (CRP), HOMA-IR, and triglyceride, signifying systemic inflammation, insulin resistance, and metabolic dysfunction associated with NAFLD progression and severity [9-11]. HOMA-IR cut-off values for insulin resistance vary across populations, ethnicities, and conditions, ranging from 2.5 to 3.0 in adults [12], and metabolic syndrome [13], to 3.8 in type 2 diabetes mellitus (T2DM) [14], 2.5-3.0 in NAFLD, and 3.0-3.5 in non-alcoholic steatohepatitis (NASH) [15]. Polycystic ovary syndrome (PCOS), cardiovascular disease (CVD) risk, obesity, and gestational diabetes mellitus (GDM) have thresholds of 2.0-2.5 [16], >2.5 [17], >2.5 [18], and >2.5 [19], respectively. Age, gender, ethnicity, and coexisting conditions should be considered when interpreting HOMA-IR. Genetic differences among studies, populations, and ethnicities result in varying HOMA-IR cut-off values, necessitating clinical judgment and additional criteria [20-24].

NAFLD progression starts with simple steatosis (NAFL), marked by over 5% fat in hepatocytes without inflammation, and can advance to non-alcoholic steatohepatitis (NASH), involving fat accumulation, inflammation, cell damage, and fibrosis (F0-F4). The most severe stage is cirrhosis, characterized by extensive scarring and liver dysfunction [20-24]. These stages guide NAFLD management and treatment approaches.

This research examines the link between insulin resistance (measured by HOMA-IR) and fatty liver diseases (NAFLD/MAFLD) in South Asian adults, investigating how insulin resistance and extrahepatic biomarkers influence disease progression and severity. The study aims to provide data for more precise and effective treatments for this specific population.

## Review

Methods

Protocol Registration

The protocol for this systematic review was registered in PROSPERO, a global repository for systematic review protocols (registration ID: CRD42024582968). The review process adhered to the Preferred Reporting Items for Systematic Reviews and Meta-Analyses (PRISMA) guidelines for conducting and reporting systematic reviews and meta-analyses [25].

Literature Search

**Figure 1:**
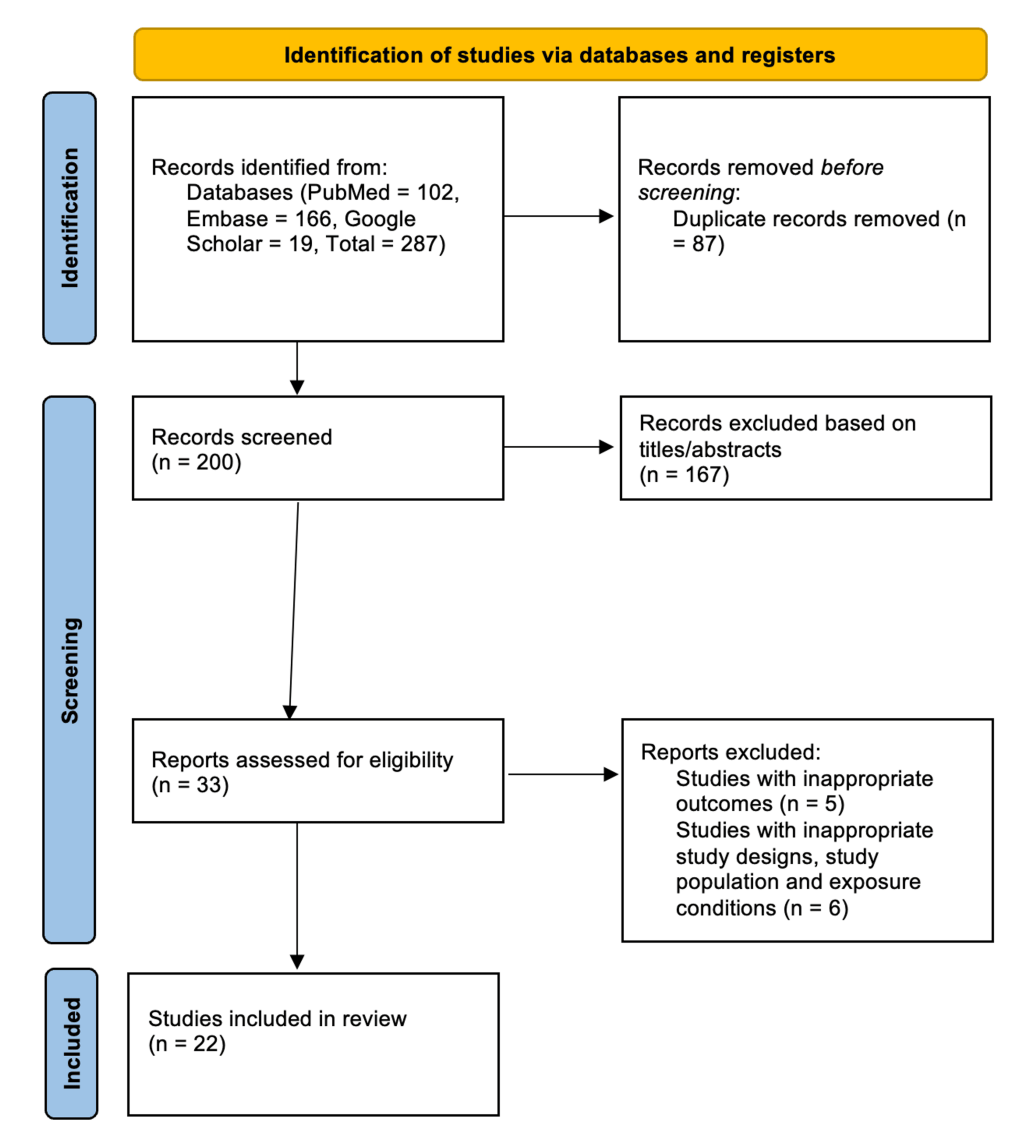
Flowchart of the study selection for the meta-analysis for the association between HOMA-IR and NAFLD/MAFLD in the South Asian population HOMA-IR: Homeostasis Model Assessment of Insulin Resistance, NAFLD: non-alcoholic fatty liver disease, MAFLD: metabolic-associated fatty liver disease

A comprehensive review of medical literature was conducted utilizing PubMed, Embase, and Google Scholar to investigate the association between Homeostasis Model Assessment of Insulin Resistance (HOMA-IR) and non-alcoholic fatty liver disease (NAFLD)/metabolic-associated fatty liver disease (MAFLD) in South Asian populations, encompassing India, Pakistan, Sri Lanka, Bangladesh, Bhutan, and the Maldives. The search terms pertained to NAFLD, MAFLD, HOMA-IR, and related concepts, with a detailed search strategy provided in the Appendices. The final search concluded on August 15, 2024. The preliminary search resulted in 287 articles in total. These were then processed using Rayyan software (Rayyan Systems Inc., Cambridge, MA) to remove duplicate entries and perform additional screening. Following the removal of 87 duplicates, 200 articles remained for additional assessment. Of these 200 articles, 167 were excluded based on titles and abstracts, leaving 33 for full-text evaluation. Eleven articles were subsequently excluded due to unsuitable study design, population, or exposure conditions (n=6) or unsuitable outcome measures (n=5), resulting in 22 studies being included in the meta-analysis. When full-text articles were unavailable, the authors were contacted through email or platforms such as ResearchGate to obtain the complete texts. The selection process is illustrated in the PRISMA flowchart (Figure 1), ensuring transparency and adherence to systematic review standards. The final selection comprised 14 case-control studies, six cross-sectional studies, and two RCTs.

Study Selection Process With Inclusion and Exclusion Criteria

A team of three independent investigators assessed articles based on predetermined criteria outlined as follows. The research incorporated publications from after 2000 that examined NAFLD/MAFLD in South Asian communities, with a restriction to English language sources. Acceptable study designs included randomized controlled trials, case-control investigations, cohort analyses, and cross-sectional surveys, with a particular emphasis on HOMA-IR and other pertinent biomarkers. Moreover, the evaluation considered non-hepatic biomarkers beyond HOMA-IR, e.g., triglyceride-glucose (TyG) and CRP levels. For specific subgroups, our study encompassed research focusing on NAFLD/MAFLD patients with concurrent conditions such as type 2 diabetes mellitus, metabolic syndrome, and obesity. These studies were incorporated provided they fulfilled our primary inclusion criteria and offered distinct data for NAFLD/MAFLD patients. We did not disqualify studies based on the presence of these comorbidities, given their frequent association with NAFLD/MAFLD in South Asian populations. However, we excluded research that solely concentrated on these conditions without providing specific NAFLD/MAFLD data. Exclusions involved participants under 18, systematic reviews, conference abstracts, grey literature, drug trials, supplementation studies, hormone therapy research, pregnant populations, or unrelated diseases. Studies on NAFLD subjects were also excluded if men consumed over 30 g of alcohol daily or women over 20 g daily. The titles and abstracts of all identified articles were independently evaluated by two authors (MM and JS) using predetermined inclusion and exclusion criteria. Any disagreements or uncertainties during the screening were resolved through consultation with the third author (UJ), who served as a mediator. This method ensured a comprehensive and impartial selection of relevant studies for our analysis. Among the selected studies, none from South Asian countries other than India could be included, and no cohort study met all the inclusion criteria.

Data Extraction, Synthesis, and Quality Assessment

Two reviewers used a standardized form to extract data, including study design, sample size, population characteristics, NAFLD/MAFLD diagnostic methods, and outcomes related to HOMA-IR and other biomarkers such as triglyceride (TyG) and C-reactive protein (CRP). All continuous variables were standardized to uniform units. When studies provided median values with ranges for continuous variables, these were converted to mean and standard deviation. Non-randomized study quality was assessed using the Newcastle-Ottawa Scale (NOS) [26], while RCTs were evaluated using the RoB 2.0 tool [27]. Non-randomized studies were classified as having low, moderate, or high risk of bias based on case definition, control selection, comparability, and exposure ascertainment. RCT risk of bias included randomization, blinding, deviation from intended interventions, missing outcome data, and selective reporting. Risk of bias assessments and critical appraisals are detailed in Tables 1-3.

**Table 1:** Newcastle-Ottawa Scale of risk assessment for the included case-control studies In the table, symbols are used to denote specific criteria: a single ★ shows that a study fulfills the criterion, while ★★ indicates exceptional adherence to the criterion. When using the NOS to evaluate case-control studies in systematic reviews, a score of 7-9 is considered low risk of bias, 4-6 is intermediate risk, and less than 4 is high risk. The NOS has a maximum score of 9, with each item scored from 1 point, except for comparability, which can score up to 2 points. NOS: Newcastle-Ottawa Scale

Sr. No.	Citation	Case definition	Case representativeness	Controls selection	Controls definition	Group comparability	Exposure ascertainment	Similarity in ascertainment	Non-response rate	Total stars awarded (out of 9)
1	Duseja et al. (2007) [28]	★	★	★	★	★★	★	★	★	9
2	Bajaj et al. (2009) [29]	★	★	★	★	★★	★	★	★	9
3	Sharma et al. (2009) [30]	★	★	★	★	★★	★	★	★	9
4	Sanal et al. (2011) [31]	★	★	★	★	★★	★	★	★	9
5	Bhatt et al. (2012) [32]	★	★	★	★	★	★	★	★	8
6	Kuppan et al. (2012) [33]	★	★	-	★	★★	★	★	★	8
7	Singh et al. (2015) [34]	★	★	★	★	★★	★	★	★	9
8	Swain et al. (2016) [35]	★	★	★	★	★★	★	★	★	9
9	Bhatt et al. (2020) [36]	★	★	★	★	★	★	★	★	8
10	Kamalraj et al. (2021) [37]	★	★	★	★	★★	★	★	★	9
11	Chawla et al. (2022) [38]	★	★	★	-	★	★	★	★	7
12	Mishra et al. (2022) [39]	★	★	★	★	★	★	★	-	6
13	Shinde et al. (2023) [40]	★	★	★	-	★	★	★	-	6
14	Sukumar et al. (2024) [41]	★	★	★	★	★★	★	★	★	9

**Table 2:** Newcastle-Ottawa Scale of risk assessment for the included cross-sectional studies A score of 7 or more stars on the NOS is considered good for cross-sectional studies: seven or more stars, good; 5-6 stars, satisfactory; and 0-4 stars, unsatisfactory. NOS: Newcastle-Ottawa Scale

Sr. No.	Citation	Representativeness of the cases	Sample size	Non-response rate	Ascertainment of the screening/surveillance tool	Comparability	Assessment of the outcome	Appropriate statistical test	Total score (out of 9)
1	Madan et al. (2006) [42]	1	0	0	2	0	1	1	5
2	Kalra et al. (2009) [43]	0	0	0	2	0	1	1	4
3	Balankhe et al. (2021) [44]	1	0	0	2	0	2	1	6
4	Jaiswal et al. (2021) [45]	0	0	0	2	0	1	1	4
5	Ravali et al. (2021) [46]	1	0	0	2	1	1	1	6
6	Bhat et al. (2013) [47]	1	0	0	2	0	1	1	5

**Table 3:** Risk of bias assessment for the included RCTs RCTs: randomized controlled trials, RoB: risk of bias, ITT: intention to treat, COVID-19: coronavirus disease 2019

Sr. No.	Citation	Bias arising from randomization process			Bias due to deviation from intended interventions			Bias due to missing outcome data		Bias in measurement of the outcome		Bias in selection of reported results		Overall risk of bias
		Random sequence generation	Allocation concealment	RoB judgment	Interventions	Blinding	RoB judgment	Attrition	RoB judgment	Blinding of outcome assessors	RoB judgment	Selective reporting	RoB judgment	
1	Arora et al. (2022) [48]	Performed	Concealed	Low risk	ITT, clearly defined groups	Open-label, assessors blinded	Low risk	High attrition due to COVID-19, reasons properly documented	Low risk	Outcome assessors blinded	Low risk	All outcomes reported	Low risk	Low risk
2	Joshi et al. (2023) [49]	Performed	Not clear	Some concerns	Clearly defined groups	Open label, blinding of assessors not mentioned	Some concerns	Lack of clarity	Some concerns	Not mentioned	Some concerns	All outcomes reported	Low risk	Some concerns

Statistical Analysis

Random effects models were employed in the meta-analyses to address inter-study variability. The study produced consolidated estimates, including mean differences for HOMA-IR, fasting blood glucose (FBG), fasting insulin, triglycerides, and CRP levels, as well as effect sizes. Analyses excluded studies lacking data for specific variables. Heterogeneity was evaluated using the I² statistic, while subgroup analyses compared outcomes such as HOMA-IR between obese and non-obese NAFLD patients. Publication bias was assessed through funnel plots. The software utilized for all analyses was Review Manager (RevMan) version 5.3 (The Cochrane Collaboration, London, UK). This program was created in 2014 by The Nordic Cochrane Centre, which is part of The Cochrane Collaboration located in Copenhagen. The research calculated pooled mean differences for various blood parameters (HOMA-IR, FBG (mg/dL), fasting insulin (µU/mL), triglyceride (mg/dL), and CRP (mg/L)) in NAFLD and healthy populations, as well as obese and non-obese groups. Random effects were measured using τ² and I² values. The I² statistic was used to assess between-study heterogeneity, quantifying the proportion of variation across studies due to genuine differences rather than chance. Heterogeneity was categorized as mild, moderate, or severe based on I² values of 25%, 50%, and 75%, respectively, using the Cochran's Q test. Funnel plots were examined to evaluate potential publication bias. Statistical significance was determined at a two-tailed P-value < 0.05.

Results

Characteristics of the Studies

The study population included 5,782 South Asian individuals with NAFLD/MAFLD (cases) and 1,366 healthy controls from non-RCT studies. The mean age was 44.6 ± 10.2 years for cases and 40.8 ± 9.4 years for controls. Males had a higher prevalence or detection rate of NAFLD/MAFLD, with 61.9% of cases and 67.4% of controls being male (Table 4).

**Table 4:** Characteristics of the participants of the studies included in the systematic review *Cases (NAFLD), **controls (healthy) NA: not available, MS: metabolic syndrome, T2DM: type 2 diabetes mellitus, NAFLD: non-alcoholic fatty liver disease, MAFLD: metabolic-associate fatty liver disease, USG: ultrasonography, MRS: magnetic resonance spectroscopy, MRI: magnetic resonance imaging, ALT: alanine aminotransferase, TE: transient elastography, BMI: body mass index The studies are listed in the table according to the year they were published.

Sr. No.	Authors	Study year	Study design	Location	Study setting	Pre-existing comorbidities in cases	Sample size	Age (years)	Gender	How NAFLD/MAFLD diagnosis was made
1	Madan et al. [42]	2006	Cross-sectional	New Delhi	Hospital-based	NA	51*	34 years*	Male: 46*, female: 5*	USG, histology
2	Duseja et al. [28]	2007	Case-control	Chandigarh	Hospital-based	MS, hypertension	22*, 15**	37 ± 10.5 years*, 39 ± 7.5 years**	Male: 15*, female: 7*; male: 10**, female: 5**	Histology
3	Bajaj et al. [29]	2009	Case-control	Allahabad	Hospital-based	MS, insulin resistance	39*, 82**	40.9 ± 11.1 years*, 35.0 ± 13.4 years**	Male: 20*, female: 19*; male: 54**, female: 28**	USG
4	Sharma et al. [30]	2009	Case-control	New Delhi	Hospital-based	NA	40*, 20**	37.4 ± 9.2 years*, 34.1 ± 6.8 years**	Male: 40*, male: 20**	USG, MRS
5	Kalra et al. [43]	2009	Cross-sectional	Chandigarh	Hospital-based	NA	10*	41 ± 9.2 years*	Male: 3*, female: 7*	USG, histology, MRI
6	Sanal et al. [31]	2011	Case-control	New Delhi	Hospital-based	NA	76*, 100**	40.05 ± 11.4 years*, 44.1 ± 18.7 years**	Male*: 62, female*: 14; male**: 80, female**: 20	USG, histology
7	Bhatt et al. [32]	2012	Case-control	New Delhi	Hospital-based	NA	162*, 173**	38.2 ± 7.0 years*, 37.1 ± 6.9 years**	NA	USG
8	Kuppan et al. [33]	2012	Case-control	Chennai	Community-based	T2DM	100*, 100**	43 ± 12.4 years*, 42 ± 12.2 years**	Male: 60*, female: 40*; male: 58**, female: 42**	USG, histology
9	Bhat et al. [47]	2013	Cross-sectional	Lucknow	Hospital-based	Metabolic syndrome, insulin resistance	150*	42.25 ± 10.5 years*	Male: 115*, female: 35*	USG + ALT (hepatic biomarker)
10	Singh et al. [34]	2015	Case-control	Cuttack	Hospital-based	Hypertension, T2DM, dyslipidemia	464*, 181**	NA	Male: 359*, female: 105*; male: 120**, female: 61**	USG, histology
11	Swain et al. [35]	2016	Case-control	Cuttack	Hospital-based	Metabolic syndrome, insulin resistance	939*, 101**	42.3 ± 10.9 years*, 38.4 ± 14.1 years**	Male: 736*, female: 203*; male: 64**, female: 37**	USG, histology
12	Bhatt et al. [36]	2020	Case-control	New Delhi	Hospital-based	NA	162*, 173**	44.3 ± 8.9 years*, 43.1 ± 9.1 years**	Male: 107*, female: 55*; male: 126**, female: 47**	USG
13	Kamalraj et al. [37]	2021	Case-control	Chennai	Hospital-based	T2DM	1625*, 210**	52.5 ± 10.4 years*, 51.1 ± 11.3 years**	Male: 892*, female: 733*; male: 114**, female: 96**	USG
14	Balankhe et al. [44]	2021	Cross-sectional	New Delhi	Community-based	NA	18*	43.4 ± 12.4 years*	Male: 12*, female: 6*	USG, TE
15	Jaiswal et al. [45]	2021	Cross-sectional	Northeast India	Hospital-based	T2DM	30*	43.33 ± 11.97 years*	Male: 26*, female: 14*	USG
16	Ravali et al. [46]	2021	Cross-sectional	Chennai	Hospital-based	T2DM, dyslipidemia, hypothyroidism, metabolic syndrome	128*	51.93 ± 10.39 years*	NA	USG
17	Chawla et al. [38]	2022	Case-control	Lucknow	Hospital-based	Hypertension, T2DM, dyslipidemia	70*, 24**	50.96 ± 11.53 years*, 43.04 ± 8.28 years**	Male: 36*, female: 34*; male: 11**, female: 13**	USG
18	Mishra et al. [39]	2022	Case-control	Berhampur	Hospital-based	Hypertension, T2DM	50*, 50**	46.50 ± 12.45 years*, 46.4 ± 12.3 years**	Male: 30*, female: 20*; male: 30**, female: 20**	USG
19	Arora et al. [48]	2022	RCT	North India	Hospital-based	Obesity	30* (with lifestyle modification), 29** (standard care)	41.1 ± 10.8 years*, 42.8 ± 10.3 years**	Male: 19*, female: 11*; male: 17**, female: 12**	USG
20	Shinde et al. [40]	2023	Case-control	Mumbai	Community-based	T2DM, obesity	100*, 100**	45.2 ± 12.1 years*, 44.8 ± 11.9 years**	Male: 58*, female: 42*; male: 55**, female: 45**	USG, TE
21	Joshi et al. [49]	2023	RCT	Four centers across India	Hospital-based	NA	233* (digital twin-enabled personalized nutrition), 86** (standard care)	NA	NA	MRI
22	Sukumar et al. [41]	2024	Case-control	New Delhi	Hospital-based	Higher BMI, IR	50*, 50**	36.34 ± 8.02 years*, 33.88 ± 7.24 years**	Male: 23*, female: 27*; male: 17**, female: 33**	USG

Pre-existing Comorbidities in NAFLD/MAFLD Cases

A review of health conditions in individuals with NAFLD/MAFLD identified type 2 diabetes mellitus (T2DM) as the most common comorbidity, present in 40.9% of studies, indicating a strong link between metabolic disorders and NAFLD/MAFLD. Hypertension was the second most prevalent, observed in 31.8% of studies, while metabolic syndrome appeared in 22.7%. Obesity and dyslipidemia were noted in 13.6% and 9.1% of studies, respectively. These findings highlight the complex relationship between NAFLD/MAFLD and various metabolic and cardiovascular disorders (Table 4).

Diagnostic Methods for NAFLD/MAFLD

NAFLD/MAFLD research utilized various diagnostic methods, with ultrasound (USG) being the most prevalent (68.2%) due to its accessibility and non-invasiveness. Histological analysis, more definitive but invasive, was employed in 22.7% of studies. Transient elastography (TE) and magnetic resonance imaging (MRI) were used in 9.1% and 4.5% of studies, respectively, highlighting the evolving nature of NAFLD/MAFLD diagnosis and the varying accessibility of diagnostic tools (Table 4).

Meta-Analysis of Biomarkers

**Figure 2:**
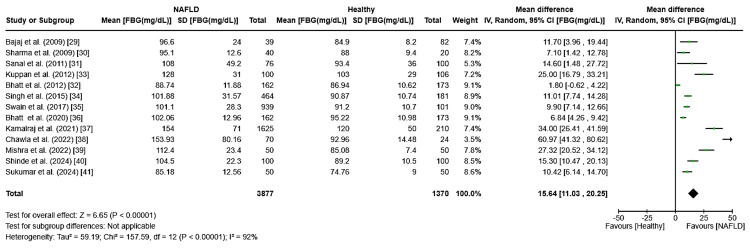
Forest plot displaying the meta-analysis of FBG levels and their association with NAFLD and healthy population for case-control studies FBG: fasting blood glucose, NAFLD: non-alcoholic fatty liver disease [29-41]

Fasting blood glucose (FBG): The meta-analysis of fasting blood glucose (FBG) levels showed a combined mean difference of 15.64 (95% CI: 11.03-20.25, I² = 92%, p < 0.0001), indicating significantly higher FBG levels in patients with NAFLD compared to healthy individuals (Figure 2). The high I² value indicates substantial variability among the studies. The study by Duseja et al. was excluded from this analysis due to missing FBG values for both groups [28].

**Figure 3:**
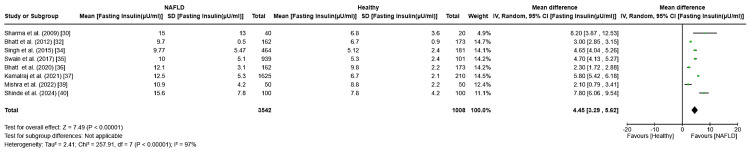
Forest plot showing the meta-analysis of fasting insulin levels and their association with NAFLD and healthy population for case-control studies NAFLD: non-alcoholic fatty liver disease [30,32,34-37,39,40]

Fasting insulin: Fasting insulin levels were significantly higher in NAFLD patients compared to healthy individuals, with a pooled mean difference of 4.45 (95% CI: 3.29-5.62, p < 0.0001). However, substantial heterogeneity was observed among the studies (I² = 97%) (Figure 3). Several studies were excluded due to incomplete group data or missing standard deviation values.

**Figure 4:**
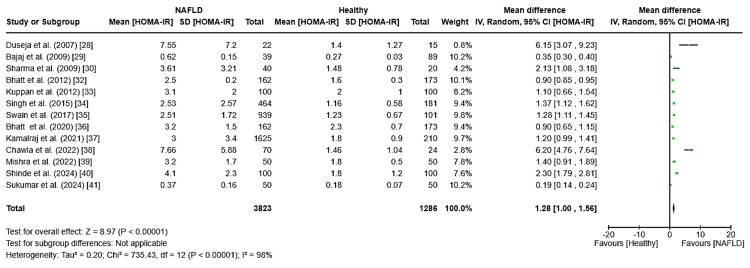
Forest plot showing the meta-analysis of HOMA-IR levels and their association with NAFLD and healthy population for case-control studies HOMA-IR: Homeostasis Model Assessment of Insulin Resistance, NAFLD: non-alcoholic fatty liver disease [28-30,32-41]

**Figure 5:**
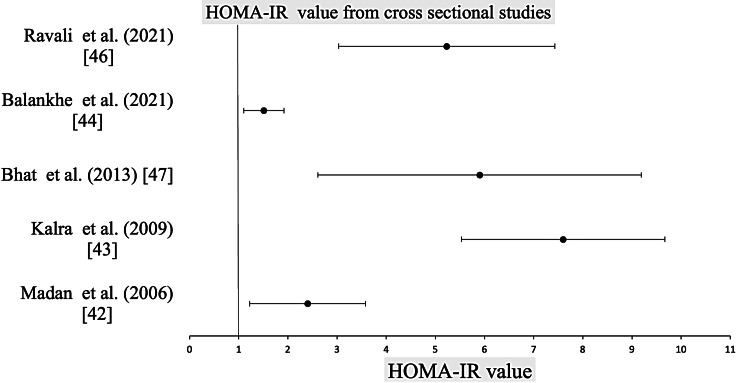
Distribution of HOMA-IR values among cross-sectional studies with NAFLD as study subjects HOMA-IR: Homeostasis Model Assessment of Insulin Resistance, NAFLD: non-alcoholic fatty liver disease [42-44,46,47]

HOMA-IR: The meta-analysis on HOMA-IR levels revealed a significant weighted mean difference (WMD) of 1.28 (95% CI: 1.00-1.58, I² = 98%, p < 0.0001) between NAFLD patients and healthy controls in case-control studies (Figure 4), indicating that higher HOMA-IR values might be linked to increased NAFLD risk. Sanal et al. was excluded due to missing control group data [31]. In cross-sectional studies, HOMA-IR mean values varied, with an overall average of 4.99 ± 1.46 among 357 NAFLD subjects. The confidence interval distribution for these HOMA-IR values in cross-sectional studies is shown in Figure 5.

**Figure 6:**
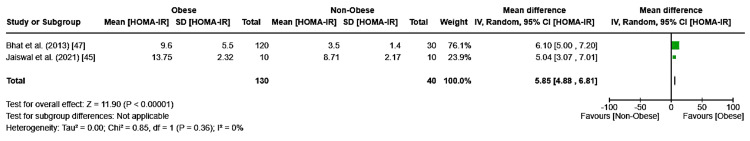
Forest plot showing the meta-analysis of HOMA-IR levels and their association between obese and non-obese patients with NAFLD in cross-sectional studies HOMA-IR: Homeostasis Model Assessment of Insulin Resistance, NAFLD: non-alcoholic fatty liver disease [45,47]

**Figure 7:**
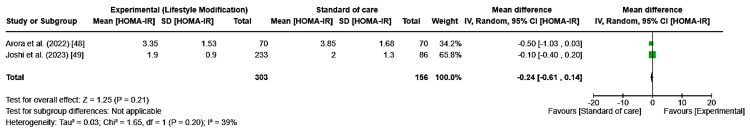
Forest plot showing the meta-analysis of HOMA-IR levels and their association with NAFLD in different intervention groups of randomized controlled trials HOMA-IR: Homeostasis Model Assessment of Insulin Resistance, NAFLD: non-alcoholic fatty liver disease [48,49]

Subgroup analysis for HOMA-IR: The weighted mean difference in HOMA-IR levels between obese and non-obese individuals with NAFLD was 5.85 (95% CI: 4.88-6.81, I² = 0%, p < 0.0001). This indicates no heterogeneity and shows that obese patients had significantly higher HOMA-IR values than non-obese patients (Figure 6). Meta-analysis of HOMA-IR levels and their association with NAFLD in different intervention groups of randomized controlled trials showed that lifestyle modification including diet changes led to lower HOMA-IR values (Figure 7). The obese NAFLD group demonstrated a mean body mass index (BMI) of 31.2 ± 3.5 kg/m², whereas the non-obese group had a mean BMI of 24.8 ± 2.1 kg/m². Furthermore, the obese group showed higher incidences of metabolic syndrome (68% versus 42%) and type 2 diabetes (35% versus 18%) in comparison to the non-obese group.

**Figure 8:**
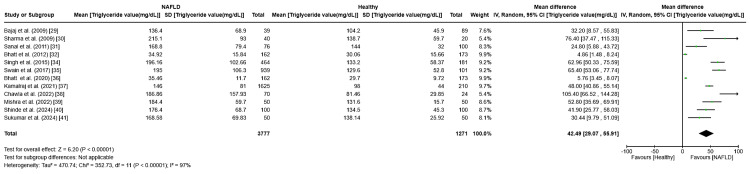
Forest plot showing the meta-analysis of triglyceride levels and their association with NAFLD and healthy population for case-control studies NAFLD: non-alcoholic fatty liver disease [29-32,34-41]

Triglycerides: Analysis revealed a pooled mean triglyceride level difference of 42.49 (95% CI: 29.07-55.91, I² = 97%, p < 0.0001), significantly elevating triglyceride levels in NAFLD patients over healthy controls. The elevated I² value indicates pronounced heterogeneity across studies (Figure 8), with exclusions from Duseja et al. [28] and Kuppan et al. [33] for lacking data.

**Figure 9:**
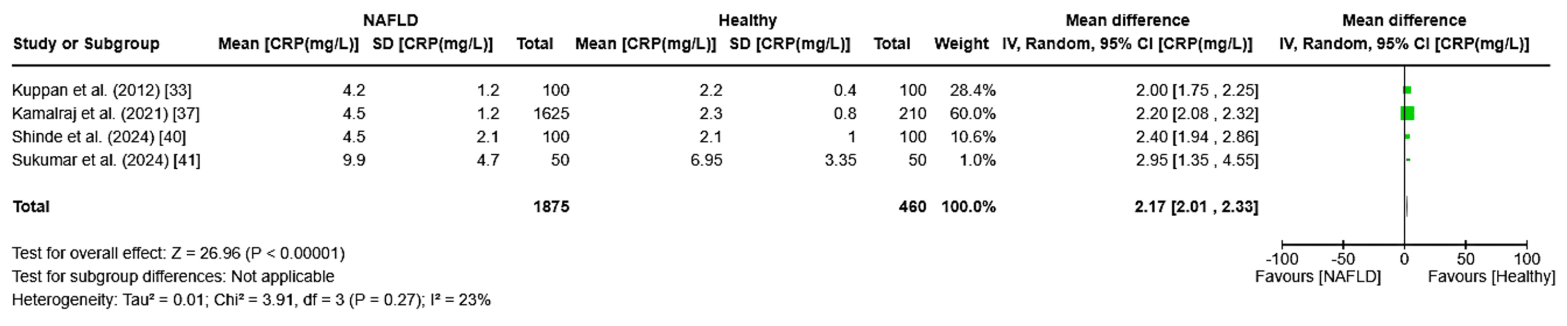
Forest plot showing the meta-analysis of CRP values and their association with NAFLD and healthy population for case-control studies CRP: C-reactive protein, NAFLD: non-alcoholic fatty liver disease [33,37,40,41]

CRP: The pooled mean difference in CRP levels was 2.17 (95% CI: 2.01-2.33, I² = 23%, p < 0.0001), indicating elevated CRP in NAFLD patients with low inter-study variability (Figure 9).

Risk of Bias Assessment

Most case-control studies had low bias risk, scoring between 7 and 9, with a few in the intermediate range (Table 1). Cross-sectional studies were rated satisfactory to good, scoring 4-6 (Table 2). Among RCTs, the study by Arora et al. had low risk across all domains [48], whereas the study by Joshi et al. had concerns due to unclear allocation concealment and lack of outcome assessor blinding (Table 3) [49].

Publication Bias and Heterogeneity

**Figure 10:**
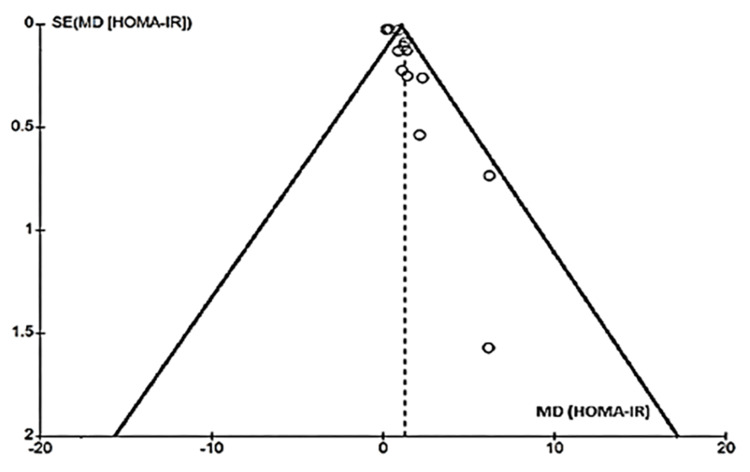
Funnel chart for publication bias (HOMA-IR) HOMA-IR: Homeostasis Model Assessment of Insulin Resistance

**Figure 11:**
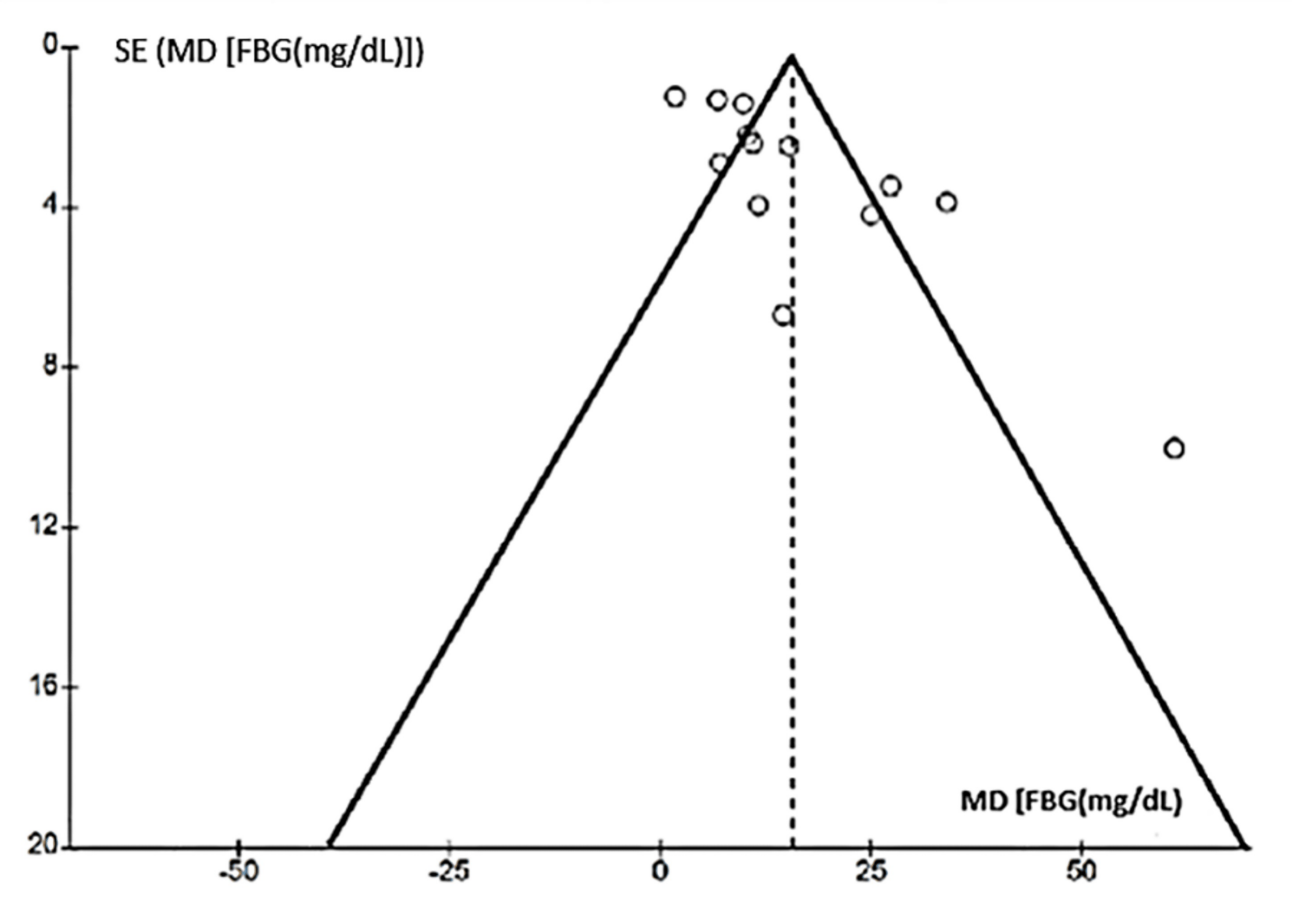
Funnel chart for publication bias (FBG) FBG: fasting blood glucose

**Figure 12:**
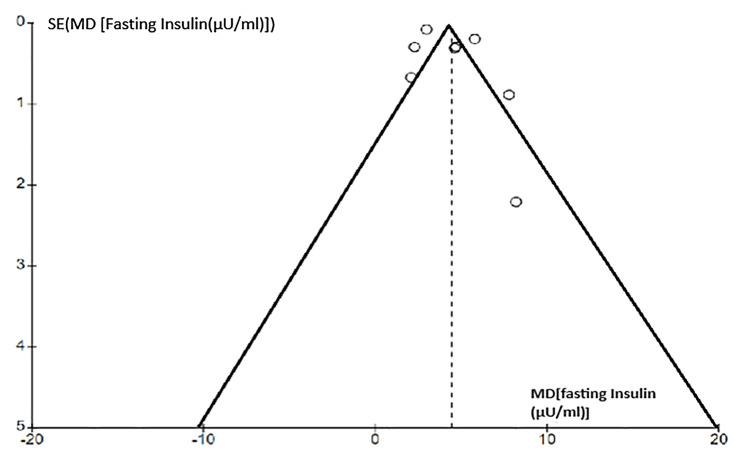
Funnel chart for publication bias (fasting insulin)

**Figure 13:**
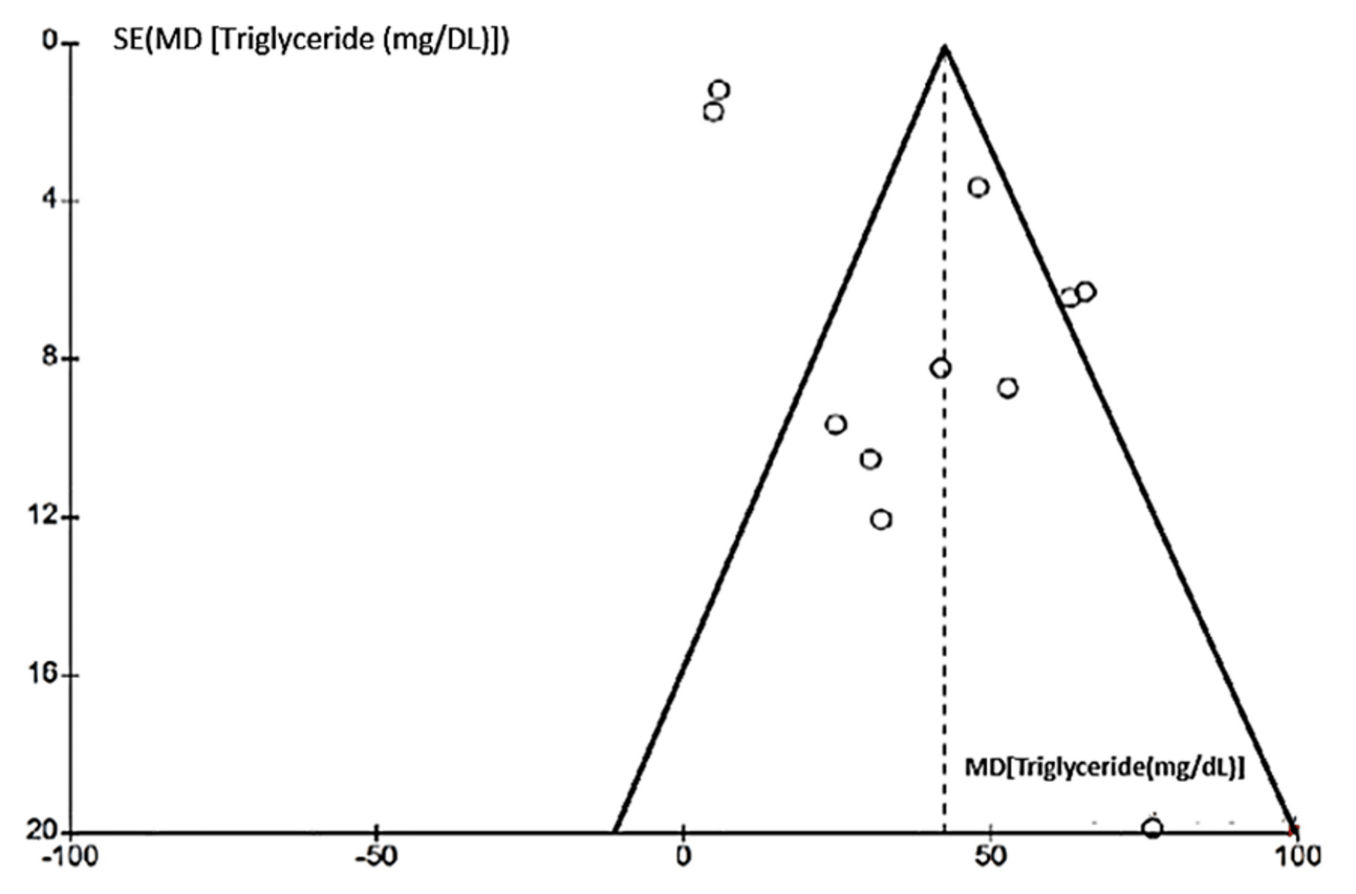
Funnel chart for publication bias (triglycerides)

Researchers examined publication bias in meta-analyses of HOMA-IR, fasting blood glucose, fasting insulin, and triglycerides using funnel plots (Figures 10-13). The plots showed no significant asymmetry, indicating a low risk of publication bias.

Quality of Evidence (Grading of Recommendations, Assessment, Development, and Evaluations (GRADE))

The GRADE methodology evaluated the quality of evidence for each outcome using the Grading of Recommendations, Assessment, Development, and Evaluations system [50]. This approach classified evidence as high, moderate, low, or very low quality by considering factors such as risk of bias, inconsistency, indirectness, imprecision, and publication bias (Table 5).

**Table 5:** GRADE evidence profile for HOMA-IR, TyG, CRP, and obese versus non-obese in NAFLD subjects GRADE: Grading of Recommendations, Assessment, Development, and Evaluations, NAFLD: non-alcoholic fatty liver disease, HOMA-IR: Homeostasis Model Assessment of Insulin Resistance, TyG: triglycerides, CRP: C-reactive protein, RCT: randomized controlled trial

Outcome	Number of studies	Study design	Risk of bias	Inconsistency	Indirectness	Imprecision	Publication bias	Quality of evidence
HOMA-IR	10 [28-34,40,48,49]	8 observational, 2 RCTs	Moderate; some studies lacked blinding or had unclear allocation concealment	High (I² = 98%) due to substantial variability in study results	No serious indirectness: populations and outcomes align with review objectives	Moderate; wide confidence intervals and variability in sample sizes	Low; funnel plots suggest minimal bias	Low to moderate
TyG	7 [35-40,48]	6 observational, 1 RCT	Low to moderate; generally well-conducted studies, some concerns about control selection	High (I² = 70%) due to differences in populations and measurement methods	No serious indirectness: studies are relevant to the review question	Moderate; some studies had small sample sizes, leading to wider confidence intervals	Minimal; publication bias not evident in funnel plot assessments	Low to moderate
CRP	8 [28-30,33,34,40,41,49]	7 observational, 1 RCT	Moderate; concerns primarily around lack of randomization and blinding in observational studies	Low (I² = 23%), indicating consistent findings across studies	No serious indirectness; outcomes relevant to clinical settings	Low; narrow confidence intervals and sufficient sample sizes	Low; broad range of study types included, reducing risk of bias	Moderate to high
Obese versus non-obese (HOMA-IR)	5 [28,32,37,40,49]	4 observational, 1 RCT	Moderate; observational studies may have selection and measurement biases	Low (I² = 0%), indicating consistent findings between subgroups	No serious indirectness; direct comparison relevant to clinical settings	Low; precise estimates with adequate sample sizes	Minimal; consistent reporting across studies	High

Discussion

The statistical analysis investigated the association between HOMA-IR, TyG, CRP, and NAFLD/MAFLD in South Asian populations, revealing significantly elevated levels of these biomarkers in affected individuals compared to healthy subjects, indicating their potential diagnostic and predictive utility.

Study Characteristics and Demographics

Table 4 outlines essential aspects of the included studies such as sample size, research design, participant characteristics, and diagnostic methods. Predominantly hospital-based, these studies focused on adults aged 40-55 years. Ultrasound, the primary diagnostic tool for NAFLD, aligns with clinical guidelines advocating non-invasive imaging for initial diagnosis. This demographic and methodological data reflects clinical diversity and offers comprehensive insights into NAFLD manifestations across various subgroups within the South Asian population.

Elevated Fasting Blood Glucose and Insulin Resistance in NAFLD

Combined data analysis showed that NAFLD patients had significantly higher fasting blood glucose (FBG) levels than healthy controls, with an average difference of 15.64 mg/dL. This aligns with global research indicating that insulin resistance, often marked by elevated FBG, is crucial in NAFLD development. Yki-Järvinen (2014) found that insulin resistance contributes to hepatic steatosis by increasing adipose tissue lipolysis, resulting in higher free fatty acid flow to the liver and enhanced de novo lipogenesis [51]. The notable heterogeneity (I² = 92%) in the results may be due to differences in study populations, including age, coexisting medical conditions, and diagnostic criteria across studies.

HOMA-IR as a Marker of Insulin Resistance

The meta-analysis uncovered a substantial increase in HOMA-IR levels among NAFLD patients when compared to healthy subjects, with a weighted mean difference of 1.28. This finding supports the notion that insulin resistance is a key factor in NAFLD development. HOMA-IR has been shown to be a dependable measure of insulin resistance, exhibiting a strong link to NAFLD risk across various populations. Research by Fan et al. (2018) indicated that a HOMA-IR above 2.5 is significantly associated with NAFLD risk in different ethnic groups, including Asians [52]. For European populations, Bedogni et al. (2005) suggested a threshold of 3.0 to differentiate NAFLD patients from healthy individuals [53]. In a multi-ethnic study, Vilar-Gomez et al. (2013) identified a cut-off of 2.7, indicating broad applicability [54]. Importantly, Kim et al. (2022) proposed a cut-off of 2.8 for South Asian populations, considering their unique metabolic risk profiles [55].

Our study, which examined HOMA-IR as a continuous variable, indicates that a HOMA-IR cut-off of roughly 2.8 might be suitable for diagnosing NAFLD in South Asian populations. This threshold is based on observed patterns in our data, particularly the notable rise in NAFLD prevalence above this level in cross-sectional studies. It is crucial to emphasize that this value emerged from data observations rather than being a pre-established cut-off. Determining a definitive threshold would require extensive sensitivity and specificity analyses in uniform cohorts, which is beyond the scope of our current meta-analysis.

The heterogeneity observed in this meta-analysis can be explained by differences in study populations, diagnostic methods, research designs, and regional variations within India. Participant characteristics and diverse diagnostic techniques contributed to outcome variability (Tables 1-3). Despite these variations, the included studies were rated as moderate to high quality, strengthening the reliability of the evidence. Additionally, funnel plot analysis showed no significant publication bias, further enhancing the credibility of the meta-analysis results. Future studies should aim to validate HOMA-IR cut-off values specifically for NAFLD/MAFLD diagnosis in South Asian populations, taking into account factors such as age, gender, and comorbidities. Such research would provide more definitive guidelines for clinical practice in this demographic.

TyG Index and Its Role in NAFLD

The meta-analysis revealed significantly elevated triglyceride-glucose (TyG) index levels in NAFLD patients, indicating its potential as a proxy for insulin resistance. Various studies have underscored the TyG index's strong correlation with NAFLD. Zhang et al. (2017) demonstrated its efficacy in detecting insulin resistance in NAFLD patients, showing high sensitivity and specificity [56]. The considerable heterogeneity observed (I² = 70%) warrants further research on lifestyle, dietary habits, and genetic predisposition affecting TyG index levels in NAFLD individuals.

CRP Levels and Systemic Inflammation in NAFLD

Studies indicate that patients with NAFLD have notably elevated levels of CRP, an indicator of systemic inflammation. Our meta-analysis demonstrated a combined mean difference in CRP levels of 2.17 mg/L (95% CI: 2.01-2.33) between NAFLD patients and healthy individuals. This aligns with worldwide evidence suggesting that ongoing inflammation contributes to NAFLD progression. According to Ridker et al. (2000), increased CRP levels may contribute to the advancement from simple steatosis to NASH [57]. The minimal heterogeneity (I² = 23%) indicates that CRP is a reliable biomarker, suggesting its potential as a therapeutic target for reducing systemic inflammation in NAFLD patients.

Subgroup Analysis: Obese Versus Non-obese NAFLD Patients

A comparative analysis of obese and non-obese NAFLD patients showed significantly higher HOMA-IR levels in the obese group, reinforcing the link between obesity, insulin resistance, and NAFLD. Chan et al. (2023) found that obesity worsens insulin resistance, hastening the transition from hepatic steatosis to severe liver conditions [58]. The lack of heterogeneity (I² = 0%) in this analysis indicates that obesity significantly affects HOMA-IR levels in NAFLD patients, highlighting the need for targeted strategies to reduce insulin resistance in obese patients and slow NAFLD progression. The significant difference in HOMA-IR levels between obese and non-obese NAFLD patients (WMD: 5.85, 95% CI: 4.88-6.81) underscores the complex interplay between obesity, insulin resistance, and NAFLD progression. Obesity, particularly visceral adiposity, is associated with increased hepatic free fatty acid flux and enhanced production of pro-inflammatory cytokines. These factors can exacerbate insulin resistance and hepatic steatosis, potentially accelerating the progression from simple steatosis to non-alcoholic steatohepatitis (NASH) and fibrosis. The higher prevalence of metabolic syndrome and type 2 diabetes in the obese NAFLD group further compounds this risk. These comorbidities are linked to more severe insulin resistance and may contribute to a more aggressive NAFLD phenotype. The presence of these metabolic abnormalities in obese NAFLD patients may necessitate more intensive management strategies, including targeted interventions to improve insulin sensitivity and reduce cardiovascular risk.

Implications for Clinical Practice and Future Research

Our research indicates that patients with NAFLD show markedly increased levels of HOMA-IR, TyG, and CRP, highlighting their potential as crucial indicators for early identification and risk assessment. Medical professionals should integrate regular evaluation of these biomarkers into their practice to enable prompt recognition of high-risk individuals who need early treatment. Upcoming studies should concentrate on establishing standardized diagnostic criteria, broadening study cohorts, conducting prospective investigations and randomized controlled trials, and investigating new risk factors. This thorough approach will not only confirm current results but also shed light on the connections between these biomarkers and NAFLD progression. Additionally, addressing variations in ethnic-specific thresholds will improve diagnostic accuracy and treatment effectiveness, significantly impacting clinical practice.

The strong link between insulin resistance and NAFLD, along with its increasing prevalence in South Asian populations, calls for targeted interventions and diagnostic approaches. We suggest the following evidence-based recommendations: (1) establish proactive HOMA-IR screening programs for high-risk individuals, especially those of South Asian origin, integrating them into primary care and national health programs; (2) create and validate risk assessment tools that incorporate HOMA-IR, TyG index, and CRP, specifically designed for South Asian populations; (3) develop culturally sensitive lifestyle modification programs addressing insulin resistance in at-risk or early-stage NAFLD patients; (4) encourage the use of non-invasive diagnostic methods, such as transient elastography combined with biomarker panels, to enhance NAFLD diagnosis and staging precision; (5) investigate early pharmacological interventions targeting insulin resistance while monitoring their effectiveness and safety in this specific population; and (6) launch public health initiatives to increase awareness about NAFLD and its risk factors within South Asian communities.

Implementing these strategies could potentially enhance early NAFLD detection, intervention efficacy, and long-term outcomes in South Asian populations. However, additional research is necessary to assess the cost-effectiveness and long-term impact of these proposed interventions.

Study limitations

This comprehensive review and statistical analysis elucidate the relationship between HOMA-IR, TyG, CRP, and NAFLD/MAFLD in South Asian populations, despite several limitations. Notably, the overrepresentation of Indian studies may limit applicability to other South Asian countries, reflecting the current research landscape and underscoring a research gap in the region.

The predominance of observational studies, especially case-control and cross-sectional designs, introduces methodological weaknesses, such as selection bias, recall bias, and uncontrolled confounding variables. For instance, case-control studies might not represent the broader population, potentially skewing the association between HOMA-IR and NAFLD. Cross-sectional studies, while useful for prevalence data, cannot establish causality or temporal links between insulin resistance and NAFLD. The lack of randomization in these studies may introduce bias in evaluating HOMA-IR levels and diagnosing NAFLD, potentially affecting pooled estimates. The heterogeneity in our meta-analysis could partially stem from these biases. Despite addressing these issues through quality assessment and sensitivity analyses, their impact cannot be entirely eliminated. The absence of randomized controlled trials (RCTs) further limits causal conclusions, reflecting broader challenges in conducting such studies in this field, particularly in South Asian populations.

Nonetheless, the moderate to high quality of included studies and minimal publication bias indicated by funnel plots suggest a degree of reliability in our findings. The observed heterogeneity underscores the need for more standardized research methodologies in future studies. Future research should focus on well-designed prospective cohort studies and RCTs across diverse South Asian populations. Such studies would provide stronger evidence for the relationship between insulin resistance markers and NAFLD, enhance the generalizability of findings, and contribute to more robust clinical guidelines for NAFLD management in South Asian populations.

## Conclusions

The meta-analysis indicates a robust link between high levels of HOMA-IR, TyG, and CRP and the occurrence of NAFLD in South Asian populations, suggesting their potential use as diagnostic indicators. Our findings propose that a HOMA-IR value of about 2.8 might be linked to higher NAFLD prevalence in this group, which aligns with earlier research and possibly reflects the distinct metabolic characteristics and risk factors of South Asian populations. However, it is important to note that this is an observational finding, not a definitively established threshold. Although our study emphasizes the significant roles of insulin resistance and inflammation in NAFLD development, additional thorough, high-quality research is required, especially in South Asian nations other than India. The consistency of our results, minimal publication bias, and the moderate to high-quality ratings of the included studies lend credibility to our conclusions. To progress the field, future studies should concentrate on standardizing diagnostic criteria and validating these biomarkers across various South Asian and global populations. This approach would improve early detection and management of NAFLD. Moreover, dedicated receiver operating characteristic (ROC) curve analyses and prospective studies are crucial for establishing and validating specific HOMA-IR thresholds for NAFLD diagnosis in South Asian populations. In summary, our findings offer valuable insights into the connection between metabolic markers and NAFLD in South Asians, establishing a foundation for enhanced diagnostic and management strategies. However, further validation and refinement of these observations are necessary to transform them into robust clinical guidelines.

## Appendices

Full search strategy (PubMed) (last searched on August 15, 2024)

Note: In our PubMed search strategy, we incorporated various MeSH terms as outlined below. The primary MeSH terms utilized were: "Non-alcoholic Fatty Liver Disease," "Insulin Resistance," "Homeostasis Model Assessment" (encompassing HOMA-IR), "Asia, Southern," terms specific to individual countries (such as "India," "Pakistan," and "Bangladesh"), terms related to study design (including "Randomized Controlled Trial," "Case-Control Studies," "Cohort Studies," and "Observational Study").

These MeSH terms were expanded to include all relevant subheadings to ensure thorough coverage. For concepts such as HOMA-IR that lack a direct MeSH term, we employed related terms such as "Homeostasis Model Assessment." The comprehensive search strategy illustrates how these MeSH terms were integrated with additional keywords and concepts to capture all relevant literature.

Search: (((((((((((((((((south asia) OR (India)) OR (sri lanka)) OR (srilanka)) OR (nepal)) OR (bangladesh)) OR (bhutan)) OR (pakistan)) OR (indian population)) OR (south asian population)) OR (sri lankan population)) OR (nepalese population)) OR (bangladeshi population)) OR (bhutanese population)) OR (pakistani population)) AND (((((HOMA) OR (HOMA-IR)) OR (Homeostasis Model Assessment-Insulin resistance)) OR (Homeostasis Model Assessment)) OR (insulin resistance))) AND (((((((((RCT) OR (rct)) OR (randomised controlled trial)) OR (randomized controlled trial)) OR (randomised trial)) OR (case-control study)) OR (case control study)) OR (cohort study)) OR (observational study))) AND ((Non-alcoholic Fatty Liver Disease[Mesh] OR Metabolic associated fatty liver disease OR MAFLD OR Metabolic-dysfunction-associated fatty liver disease OR Non alcoholic Fatty Liver Disease OR NAFLD OR Nonalcoholic Fatty Liver Disease OR Fatty Liver, Nonalcoholic OR Fatty Livers, Nonalcoholic OR Liver, Nonalcoholic Fatty OR Livers, Nonalcoholic Fatty OR Nonalcoholic Fatty Liver OR Nonalcoholic Fatty Livers OR Nonalcoholic Steatohepatitis OR Nonalcoholic Steatohepatitides OR Steatohepatitides, Nonalcoholic OR Steatohepatitis, Nonalcoholic) AND (NAFLD))

(((((((((((((((((south asia) OR (India)) OR (sri lanka)) OR (srilanka)) OR (nepal)) OR (bangladesh)) OR (bhutan)) OR (pakistan)) OR (indian population)) OR (south asian population)) OR (sri lankan population)) OR (nepalese population)) OR (bangladeshi population)) OR (bhutanese population)) OR (pakistani population)) AND (((((HOMA) OR (HOMA-IR)) OR (Homeostasis Model Assessment-Insulin resistance)) OR (Homeostasis Model Assessment)) OR (insulin resistance))) AND (((((((((RCT) OR (rct)) OR (randomised controlled trial)) OR (randomized controlled trial)) OR (randomised trial)) OR (case-control study)) OR (case control study)) OR (cohort study)) OR (observational study))) AND ((Non-alcoholic Fatty Liver Disease[Mesh] OR Metabolic associated fatty liver disease OR MAFLD OR Metabolic-dysfunction-associated fatty liver disease OR Non alcoholic Fatty Liver Disease OR NAFLD OR Nonalcoholic Fatty Liver Disease OR Fatty Liver, Nonalcoholic OR Fatty Livers, Nonalcoholic OR Liver, Nonalcoholic Fatty OR Livers, Nonalcoholic Fatty OR Nonalcoholic Fatty Liver OR Nonalcoholic Fatty Livers OR Nonalcoholic Steatohepatitis OR Nonalcoholic Steatohepatitides OR Steatohepatitides, Nonalcoholic OR Steatohepatitis, Nonalcoholic) AND (NAFLD))

Translations

south asia: "asia, southern"[MeSH Terms] OR ("asia"[All Fields] AND "southern"[All Fields]) OR "southern asia"[All Fields] OR ("south"[All Fields] AND "asia"[All Fields]) OR "south asia"[All Fields]

India: "india"[MeSH Terms] OR "india"[All Fields] OR "india's"[All Fields] OR "indias"[All Fields]

sri lanka: "sri lanka"[MeSH Terms] OR ("sri"[All Fields] AND "lanka"[All Fields]) OR "sri lanka"[All Fields]

nepal: "nepal"[MeSH Terms] OR "nepal"[All Fields] OR "nepal's"[All Fields]

bangladesh: "bangladesh"[MeSH Terms] OR "bangladesh"[All Fields] OR "bangladesh's"[All Fields]

bhutan: "bhutan"[MeSH Terms] OR "bhutan"[All Fields] OR "bhutan's"[All Fields]

pakistan: "pakistan"[MeSH Terms] OR "pakistan"[All Fields] OR "pakistan's"[All Fields]

indian: "indian"[All Fields] OR "indian's"[All Fields] OR "indians"[All Fields]

population: "populate"[All Fields] OR "populated"[All Fields] OR "populates"[All Fields] OR "populating"[All Fields] OR "population"[MeSH Terms] OR "population"[All Fields] OR "population groups"[MeSH Terms] OR ("population"[All Fields] AND "groups"[All Fields]) OR "population groups"[All Fields] OR "populations"[All Fields] OR "population's"[All Fields] OR "populational"[All Fields] OR "populations's"[All Fields] OR "populous"[All Fields]

south asian: "south asian people"[MeSH Terms] OR ("south"[All Fields] AND "asian"[All Fields] AND "people"[All Fields]) OR "south asian people"[All Fields] OR ("south"[All Fields] AND "asian"[All Fields]) OR "south asian"[All Fields]

population: "populate"[All Fields] OR "populated"[All Fields] OR "populates"[All Fields] OR "populating"[All Fields] OR "population"[MeSH Terms] OR "population"[All Fields] OR "population groups"[MeSH Terms] OR ("population"[All Fields] AND "groups"[All Fields]) OR "population groups"[All Fields] OR "populations"[All Fields] OR "population's"[All Fields] OR "populational"[All Fields] OR "populations's"[All Fields] OR "populous"[All Fields]

lankan: "lankan"[All Fields] OR "lankans"[All Fields]

population: "populate"[All Fields] OR "populated"[All Fields] OR "populates"[All Fields] OR "populating"[All Fields] OR "population"[MeSH Terms] OR "population"[All Fields] OR "population groups"[MeSH Terms] OR ("population"[All Fields] AND "groups"[All Fields]) OR "population groups"[All Fields] OR "populations"[All Fields] OR "population's"[All Fields] OR "populational"[All Fields] OR "populations's"[All Fields] OR "populous"[All Fields]

population: "populate"[All Fields] OR "populated"[All Fields] OR "populates"[All Fields] OR "populating"[All Fields] OR "population"[MeSH Terms] OR "population"[All Fields] OR "population groups"[MeSH Terms] OR ("population"[All Fields] AND "groups"[All Fields]) OR "population groups"[All Fields] OR "populations"[All Fields] OR "population's"[All Fields] OR "populational"[All Fields] OR "populations's"[All Fields] OR "populous"[All Fields]

bangladeshi: "bangladeshi"[All Fields] OR "bangladeshis"[All Fields]

population: "populate"[All Fields] OR "populated"[All Fields] OR "populates"[All Fields] OR "populating"[All Fields] OR "population"[MeSH Terms] OR "population"[All Fields] OR "population groups"[MeSH Terms] OR ("population"[All Fields] AND "groups"[All Fields]) OR "population groups"[All Fields] OR "populations"[All Fields] OR "population's"[All Fields] OR "populational"[All Fields] OR "populations's"[All Fields] OR "populous"[All Fields]

population: "populate"[All Fields] OR "populated"[All Fields] OR "populates"[All Fields] OR "populating"[All Fields] OR "population"[MeSH Terms] OR "population"[All Fields] OR "population groups"[MeSH Terms] OR ("population"[All Fields] AND "groups"[All Fields]) OR "population groups"[All Fields] OR "populations"[All Fields] OR "population's"[All Fields] OR "populational"[All Fields] OR "populations's"[All Fields] OR "populous"[All Fields]

pakistani: "Pakistani people"[Supplementary Concept] OR "Pakistani people"[All Fields] OR "pakistanis"[All Fields] OR "pakistani"[All Fields]

population: "populate"[All Fields] OR "populated"[All Fields] OR "populates"[All Fields] OR "populating"[All Fields] OR "population"[MeSH Terms] OR "population"[All Fields] OR "population groups"[MeSH Terms] OR ("population"[All Fields] AND "groups"[All Fields]) OR "population groups"[All Fields] OR "populations"[All Fields] OR "population's"[All Fields] OR "populational"[All Fields] OR "populations's"[All Fields] OR "populous"[All Fields]

Homeostasis: "homoeostasis"[All Fields] OR "homeostasis"[MeSH Terms] OR "homeostasis"[All Fields]

Model: "model"[All Fields] OR "model's"[All Fields] OR "modeled"[All Fields] OR "modeler"[All Fields] OR "modeler's"[All Fields] OR "modelers"[All Fields] OR "modeling"[All Fields] OR "modelings"[All Fields] OR "modelization"[All Fields] OR "modelizations"[All Fields] OR "modelize"[All Fields] OR "modelized"[All Fields] OR "modelled"[All Fields] OR "modeller"[All Fields] OR "modellers"[All Fields] OR "modelling"[All Fields] OR "modellings"[All Fields] OR "models"[All Fields]

resistance: "resist"[All Fields] OR "resistance"[All Fields] OR "resistances"[All Fields] OR "resistant"[All Fields] OR "resistants"[All Fields] OR "resisted"[All Fields] OR "resistence"[All Fields] OR "resistences"[All Fields] OR "resistent"[All Fields] OR "resistibility"[All Fields] OR "resisting"[All Fields] OR "resistive"[All Fields] OR "resistively"[All Fields] OR "resistivities"[All Fields] OR "resistivity"[All Fields] OR "resists"[All Fields]

Homeostasis: "homoeostasis"[All Fields] OR "homeostasis"[MeSH Terms] OR "homeostasis"[All Fields]

Model: "model"[All Fields] OR "model's"[All Fields] OR "modeled"[All Fields] OR "modeler"[All Fields] OR "modeler's"[All Fields] OR "modelers"[All Fields] OR "modeling"[All Fields] OR "modelings"[All Fields] OR "modelization"[All Fields] OR "modelizations"[All Fields] OR "modelize"[All Fields] OR "modelized"[All Fields] OR "modelled"[All Fields] OR "modeller"[All Fields] OR "modellers"[All Fields] OR "modelling"[All Fields] OR "modellings"[All Fields] OR "models"[All Fields]

Assessment: "assess"[All Fields] OR "assessed"[All Fields] OR "assessement"[All Fields] OR "assesses"[All Fields] OR "assessing"[All Fields] OR "assessment"[All Fields] OR "assessment's"[All Fields] OR "assessments"[All Fields]

insulin resistance: "insulin resistance"[MeSH Terms] OR ("insulin"[All Fields] AND "resistance"[All Fields]) OR "insulin resistance"[All Fields]

randomised controlled trial: "randomized controlled trial"[Publication Type] OR "randomized controlled trials as topic"[MeSH Terms] OR "randomised controlled trial"[All Fields] OR "randomized controlled trial"[All Fields]

randomized controlled trial: "randomized controlled trial"[Publication Type] OR "randomized controlled trials as topic"[MeSH Terms] OR "randomized controlled trial"[All Fields] OR "randomised controlled trial"[All Fields]

randomised: "random allocation"[MeSH Terms] OR ("random"[All Fields] AND "allocation"[All Fields]) OR "random allocation"[All Fields] OR "randomization"[All Fields] OR "randomized"[All Fields] OR "random"[All Fields] OR "randomisation"[All Fields] OR "randomisations"[All Fields] OR "randomise"[All Fields] OR "randomised"[All Fields] OR "randomising"[All Fields] OR "randomizations"[All Fields] OR "randomize"[All Fields] OR "randomizes"[All Fields] OR "randomizing"[All Fields] OR "randomness"[All Fields] OR "randoms"[All Fields]

trial: "clinical trials as topic"[MeSH Terms] OR ("clinical"[All Fields] AND "trials"[All Fields] AND "topic"[All Fields]) OR "clinical trials as topic"[All Fields] OR "trial"[All Fields] OR "trial's"[All Fields] OR "trialed"[All Fields] OR "trialing"[All Fields] OR "trials"[All Fields]

case-control study: "case-control studies"[MeSH Terms] OR ("case-control"[All Fields] AND "studies"[All Fields]) OR "case-control studies"[All Fields] OR ("case"[All Fields] AND "control"[All Fields] AND "study"[All Fields]) OR "case control study"[All Fields]

case control study: "case-control studies"[MeSH Terms] OR ("case-control"[All Fields] AND "studies"[All Fields]) OR "case-control studies"[All Fields] OR ("case"[All Fields] AND "control"[All Fields] AND "study"[All Fields]) OR "case control study"[All Fields]

cohort study: "cohort studies"[MeSH Terms] OR ("cohort"[All Fields] AND "studies"[All Fields]) OR "cohort studies"[All Fields] OR ("cohort"[All Fields] AND "study"[All Fields]) OR "cohort study"[All Fields]

observational study: "observational study"[Publication Type] OR "observational studies as topic"[MeSH Terms] OR "observational study"[All Fields]

Non-alcoholic Fatty Liver Disease[Mesh]: "non-alcoholic fatty liver disease"[MeSH Terms]

Metabolic: "metabolic"[All Fields] OR "metabolical"[All Fields] OR "metabolically"[All Fields] OR "metabolics"[All Fields] OR "metabolism"[MeSH Terms] OR "metabolism"[All Fields] OR "metabolisms"[All Fields] OR "metabolism"[Subheading] OR "metabolities"[All Fields] OR "metabolization"[All Fields] OR "metabolize"[All Fields] OR "metabolized"[All Fields] OR "metabolizer"[All Fields] OR "metabolizers"[All Fields] OR "metabolizes"[All Fields] OR "metabolizing"[All Fields]

associated: "associate"[All Fields] OR "associated"[All Fields] OR "associates"[All Fields] OR "associating"[All Fields] OR "association"[MeSH Terms] OR "association"[All Fields] OR "associations"[All Fields]

fatty liver disease: "non-alcoholic fatty liver disease"[MeSH Terms] OR ("non-alcoholic"[All Fields] AND "fatty"[All Fields] AND "liver"[All Fields] AND "disease"[All Fields]) OR "non-alcoholic fatty liver disease"[All Fields] OR ("fatty"[All Fields] AND "liver"[All Fields] AND "disease"[All Fields]) OR "fatty liver disease"[All Fields]

fatty liver disease: "non-alcoholic fatty liver disease"[MeSH Terms] OR ("non-alcoholic"[All Fields] AND "fatty"[All Fields] AND "liver"[All Fields] AND "disease"[All Fields]) OR "non-alcoholic fatty liver disease"[All Fields] OR ("fatty"[All Fields] AND "liver"[All Fields] AND "disease"[All Fields]) OR "fatty liver disease"[All Fields]

Non alcoholic Fatty Liver Disease: "non-alcoholic fatty liver disease"[MeSH Terms] OR ("non-alcoholic"[All Fields] AND "fatty"[All Fields] AND "liver"[All Fields] AND "disease"[All Fields]) OR "non-alcoholic fatty liver disease"[All Fields] OR ("non"[All Fields] AND "alcoholic"[All Fields] AND "fatty"[All Fields] AND "liver"[All Fields] AND "disease"[All Fields]) OR "non alcoholic fatty liver disease"[All Fields]

NAFLD: "naflds"[All Fields] OR "non-alcoholic fatty liver disease"[MeSH Terms] OR ("non-alcoholic"[All Fields] AND "fatty"[All Fields] AND "liver"[All Fields] AND "disease"[All Fields]) OR "non-alcoholic fatty liver disease"[All Fields] OR "nafld"[All Fields]

Nonalcoholic Fatty Liver Disease: "non-alcoholic fatty liver disease"[MeSH Terms] OR ("non-alcoholic"[All Fields] AND "fatty"[All Fields] AND "liver"[All Fields] AND "disease"[All Fields]) OR "non-alcoholic fatty liver disease"[All Fields] OR ("nonalcoholic"[All Fields] AND "fatty"[All Fields] AND "liver"[All Fields] AND "disease"[All Fields]) OR "nonalcoholic fatty liver disease"[All Fields]

Fatty Liver, Nonalcoholic: "non-alcoholic fatty liver disease"[MeSH Terms] OR ("non-alcoholic"[All Fields] AND "fatty"[All Fields] AND "liver"[All Fields] AND "disease"[All Fields]) OR "non-alcoholic fatty liver disease"[All Fields] OR ("fatty"[All Fields] AND "liver"[All Fields] AND "nonalcoholic"[All Fields]) OR "fatty liver, nonalcoholic"[All Fields]

Fatty Livers, Nonalcoholic: "non-alcoholic fatty liver disease"[MeSH Terms] OR ("non-alcoholic"[All Fields] AND "fatty"[All Fields] AND "liver"[All Fields] AND "disease"[All Fields]) OR "non-alcoholic fatty liver disease"[All Fields] OR ("fatty"[All Fields] AND "livers"[All Fields] AND "nonalcoholic"[All Fields]) OR "fatty livers, nonalcoholic"[All Fields]

Liver, Nonalcoholic Fatty: "non-alcoholic fatty liver disease"[MeSH Terms] OR ("non-alcoholic"[All Fields] AND "fatty"[All Fields] AND "liver"[All Fields] AND "disease"[All Fields]) OR "non-alcoholic fatty liver disease"[All Fields] OR ("liver"[All Fields] AND "nonalcoholic"[All Fields] AND "fatty"[All Fields]) OR "liver, nonalcoholic fatty"[All Fields]

Livers, Nonalcoholic Fatty: "non-alcoholic fatty liver disease"[MeSH Terms] OR ("non-alcoholic"[All Fields] AND "fatty"[All Fields] AND "liver"[All Fields] AND "disease"[All Fields]) OR "non-alcoholic fatty liver disease"[All Fields] OR ("livers"[All Fields] AND "nonalcoholic"[All Fields] AND "fatty"[All Fields]) OR "livers, nonalcoholic fatty"[All Fields]

Nonalcoholic Fatty Liver: "non-alcoholic fatty liver disease"[MeSH Terms] OR ("non-alcoholic"[All Fields] AND "fatty"[All Fields] AND "liver"[All Fields] AND "disease"[All Fields]) OR "non-alcoholic fatty liver disease"[All Fields] OR ("nonalcoholic"[All Fields] AND "fatty"[All Fields] AND "liver"[All Fields]) OR "nonalcoholic fatty liver"[All Fields]

Nonalcoholic Fatty Livers: "non-alcoholic fatty liver disease"[MeSH Terms] OR ("non-alcoholic"[All Fields] AND "fatty"[All Fields] AND "liver"[All Fields] AND "disease"[All Fields]) OR "non-alcoholic fatty liver disease"[All Fields] OR ("nonalcoholic"[All Fields] AND "fatty"[All Fields] AND "livers"[All Fields]) OR "nonalcoholic fatty livers"[All Fields]

Nonalcoholic Steatohepatitis: "non-alcoholic fatty liver disease"[MeSH Terms] OR ("non-alcoholic"[All Fields] AND "fatty"[All Fields] AND "liver"[All Fields] AND "disease"[All Fields]) OR "non-alcoholic fatty liver disease"[All Fields] OR ("nonalcoholic"[All Fields] AND "steatohepatitis"[All Fields]) OR "nonalcoholic steatohepatitis"[All Fields]

Nonalcoholic Steatohepatitides: "non-alcoholic fatty liver disease"[MeSH Terms] OR ("non-alcoholic"[All Fields] AND "fatty"[All Fields] AND "liver"[All Fields] AND "disease"[All Fields]) OR "non-alcoholic fatty liver disease"[All Fields] OR ("nonalcoholic"[All Fields] AND "steatohepatitides"[All Fields]) OR "nonalcoholic steatohepatitides"[All Fields]

Steatohepatitides, Nonalcoholic: "non-alcoholic fatty liver disease"[MeSH Terms] OR ("non-alcoholic"[All Fields] AND "fatty"[All Fields] AND "liver"[All Fields] AND "disease"[All Fields]) OR "non-alcoholic fatty liver disease"[All Fields] OR ("steatohepatitides"[All Fields] AND "nonalcoholic"[All Fields]) OR "steatohepatitides, nonalcoholic"[All Fields]

Steatohepatitis, Nonalcoholic: "non-alcoholic fatty liver disease"[MeSH Terms] OR ("non-alcoholic"[All Fields] AND "fatty"[All Fields] AND "liver"[All Fields] AND "disease"[All Fields]) OR "non-alcoholic fatty liver disease"[All Fields] OR ("steatohepatitis"[All Fields] AND "nonalcoholic"[All Fields]) OR "steatohepatitis, nonalcoholic"[All Fields]

NAFLD: "naflds"[All Fields] OR "non-alcoholic fatty liver disease"[MeSH Terms] OR ("non-alcoholic"[All Fields] AND "fatty"[All Fields] AND "liver"[All Fields] AND "disease"[All Fields]) OR "non-alcoholic fatty liver disease"[All Fields] OR "nafld"[All Fields]

Embase search history

Embase (1996 to August 13, 2024)

((south asia or India or sri lanka or srilanka or nepal or bangladesh or bhutan or pakistan or Indian population or south asian population or sri lankan population or nepalese population or bangladeshi population or bhutanese population or pakistani population) and (HOMA or HOMA-IR or Homeostasis Model Assessment-Insulin resistance or Homeostasis Model Assessment or insulin resistance) and (RCT or rct or randomised controlled trial or randomized controlled trial or randomised trial or case-control study or case control study or cohort study or observational study) and (Non-alcoholic Fatty Liver Disease or Metabolic associated fatty liver disease or MAFLD or Metabolic-dysfunction-associated fatty liver disease or Non alcoholic Fatty Liver Disease or NAFLD or Nonalcoholic Fatty Liver Disease or Fatty Liver, Nonalcoholic or Fatty Livers, Nonalcoholic or Liver, Nonalcoholic Fatty or Livers, Nonalcoholic Fatty or Nonalcoholic Fatty Liver or Nonalcoholic Fatty Livers or Nonalcoholic Steatohepatitis or Nonalcoholic Steatohepatitides or Steatohepatitides Nonalcoholic or Steatohepatitis)).af.

Google Scholar search strategy (Last searched on August 15, 2023)

We adjusted our search approach due to the platform's limitations in handling complex Boolean logic and its lack of a controlled vocabulary similar to MeSH. We employed simplified search strings based on key concepts from our PubMed and Embase strategies. Our basic Google Scholar search structure was as follows: (NAFLD OR "fatty liver") AND (HOMA-IR OR "insulin resistance") AND ("South Asia" OR India OR Pakistan OR Bangladesh OR "Sri Lanka" OR Nepal OR Bhutan).

We examined the top 200 results, ranked by relevance, to identify potentially eligible studies. This Google Scholar approach complemented our more structured searches in PubMed and Embase, helping to uncover additional relevant literature, particularly recent publications, which were conducted in South Asian populations that might not yet be indexed in the other databases. The findings from Google Scholar were then merged with those from PubMed and Embase for the screening process, as outlined in our PRISMA flow diagram.
